# Walking Improves Cardiac Function: A Trial on the Effects of Walking on Left Ventricular Function in Type 2 Diabetes Patients

**DOI:** 10.3390/jcdd13030136

**Published:** 2026-03-12

**Authors:** Roman Leischik, Patrick Bank, Christian Erik Gerlach, Fabian Sanchis-Gomar

**Affiliations:** 1 Department of Cardiology, Section Prevention and Sports Medicine, Faculty of Health, School of Medicine, University Witten/Herdecke, 58455 Witten, Germany; patrick.bank@ruhr-uni-bochum.de (P.B.); ceegerlach@goolemail.com (C.E.G.); 2 Division of Cardiovascular Medicine, Stanford University School of Medicine, Stanford, CA 94305, USA; fabian.sanchis@uv.es

**Keywords:** walking intervention, walking, type 2 diabetes, exercise, cardiac function, physical activity

## Abstract

Cardiometabolic abnormalities, which are common in diabetes patients, can be alleviated through exercise. We examined the specific effects of walking (4–5 METS) on diabetic patients’ cardiac function in a randomized study. Patients with type 2 diabetes (metformin-, insulin-, and diet-controlled; n = 32) were randomized to a 12-week walking intervention (40 min, three times/week; n = 16) or standard care (control group, n = 16). We prospectively compared metabolic, anthropometric, cardiac function and cardiorespiratory fitness parameters between the two groups via linear regression. Compared with that of the control group, the postintervention global strain of the walking group improved significantly (−19.0 (±3.0) vs. −20.9 (±2.6), Diff = −1.92 (CI = −2.61–−1.24), *p* < 0.001; control: −18.7 (±3.2) vs. −18.9 (±3.6), Diff = −0.19 (CI = −1.00–0.63), *p* = 0.650), with a pre/post between-group estimated mean difference of ~−1.73 (CI = −2.78–−0.69; *p* < 0.001). Abdominal circumference (−3 cm (CI = −4.41–−1.59), *p* < 0.001)), resting heart rate/bpm (−6.50 (CI = −9.69–−3.31, *p* < 0.001)) and body fat percentage (−2.74 (CI = −4.71–−0.76, *p* < 0.007)) changed significantly only in the walking group. Spiroergometric data revealed improved oxygen uptake in the walking group vs. the control group: abs_VO2max/L·min^−1^ (0.19 (0.05–0.33), *p* < 0.008); rel_VO2max/mL·kg^−1^·min^−1^ (2.43 (1.03–3.83), *p* < 0.001). This first randomized intervention study of supervised walking in patients with type 2 diabetes demonstrated that even moderate-intensity physical activity (such as walking) can improve cardiac function and body composition, reduced waist circumference, and increased oxygen uptake, making it a cost-effective treatment with significant preventive and restorative benefits for cardiac function and body composition in these patients.

## 1. Introduction

Physical activity (PA) is among the most important internal medicine therapies [1]; specifically, exercise training, including intensive training for elite athletes, can lead to myocardial adaptation [2], with negative cardiac consequences occurring only in the presence of adverse genetic predisposing factors or inflammation [2].

Insufficient PA is the greatest health problem of the 21st century [3]. Diabetes mellitus (DM) is currently the most significant [4,5], as it will affect almost a billion people in the coming years [5,6,7]. One of the many causes of obesity, diabetes, and metabolic syndrome is a lack of exercise [8,9,10], sugar intake [11], and social circumstances [12]. Generally, increasing PA leads to a reduction in the incidence of chronic diseases and improvements in overall public health [13]. Compared with nondiabetic patients, diabetic patients have a greater incidence of heart disease, including an increased incidence and severity of congestive heart failure [14]. Walking is a low-cost and preventive intervention for patients with DM [15,16].

In the general population, even 30 min of daily physical activity is associated with a reduced risk of mortality [17], a lower likelihood of depression [18], improved weight control, and a reduced risk of colon cancer [19]. In patients with type 2 DM (T2DM) and diabetic cardiomyopathy (DCM), several reviews have described positive effects of exercise on cardiac function, particularly regarding diastolic function and beneficial remodelling, but not necessarily consistent improvements in systolic function or left ventricular structure, regardless of exercise type [20,21,22]. Exercise has been shown to improve cardiac function in diabetic animals [23] and diastolic function in humans [24]; however, the effects of exercise on systolic function remain controversial [20,22,25] and may depend on exercise modality and intensity (i.e., whether moderate-intensity exercise can also improve cardiac function compared with high-intensity exercise) [25].

Thee study was fundamentally designed to investigate the effects of 12 weeks of moderate exercise (walking) in patients with type 2 diabetes, particularly with respect to body composition, cardiac function, and cognition. The analysis of cardiac function was an important part of the study, and one doctoral student focused specifically on this aspect. The study endpoint was defined as the completion of the 12-week physical training period.

Therefore, we hypothesized that a 12-week group-based walking program would lead to measurable improvements in cardiac function, with a particular focus on strain-derived systolic parameters, in patients with type 2 diabetes.

Only four prospective and randomized studies on the impact of exercise on systolic cardiac function in patients with diabetes have been conducted thus far (the first study was published in three parts with the same design [25,26,27], and the last three [28,29,30] evaluated high-intensity interval training (HIIT)). The cited moderate-intensity exercise studies were conducted between 2009 and 2019 in patients with T2DM. The first cited study evaluated the effects of only a 4-week supervised, gym-based programme in which participants attempted to achieve a minimum of 150 min of moderate-intensity exercise a week for three months [25,26,27]. These publications (two of which were based on the same patient cohort and design) did not identify improvements in systolic myocardial function in diabetic patients. The three other cited studies, published by Hollekim-Strand et al. [29], Cassidy et al. [28] and Wilson et al. [30], evaluated the effects of HIIT. Cassidy et al. [28] included 12 patients and 11 control participants and demonstrated an improvement in cardiac function after 12 weeks of HIIT. Hollekim-Strand et al. [29] reported an improvement in global strain after 12 weeks of HIIT without changes in body fat percentage. Wilson et al. [30] examined 11 patients with T2DM and 5 control participants and did not observe any changes in systolic function (ejection fraction) with HIIT. These studies of different types of physical exercise of varying intensity demonstrated diverse results, with some showing improvement in systolic cardiac function and others showing no change. However, the effect of supervised walking alone on echocardiographic measures of cardiac function in patients with type 2 diabetes has not yet been evaluated in a randomized controlled trial.

We examined the specific effects of walking on the cardiac function of diabetic patients in a randomized study to determine whether moderate physical exercise (4–5 METS) can produce improvements in this patient population.

## 2. Methods

### 2.1. Study Population

The study cohort consisted of overweight and obese male and female patients with type 2 diabetes from the Hagen City area.

Potential participants were identified through the practice management system of Professor Leischik and colleagues in Hagen (Hagen, Germany). Their willingness to participate was initially assessed by telephone. Additional recruitment strategies included an article in the weekly newspaper Hagener Wochenkurier, study flyers displayed in specialist practices and hospitals in Hagen, and information provided through an online study presence. Participants could contact the study team via a dedicated study mobile phone and a specific email address. Appointments for baseline and follow-up examinations were arranged either in person or through digital communication channels. Inclusion criteria: BMI > 28, Type 2 diabetes mellitus, diet-controlled or insulin-treated, age 35–75 years, and written informed consent after detailed explanation. Exclusion criteria: Diseases impairing myocardial function, including myocardial infarction within the previous 6 weeks, heart failure NYHA class III–IV, hemodynamically relevant valvular disease or decompensated aortic stenosis, severe arterial hypertension (grade III; systolic > 180 mmHg and/or diastolic > 110 mmHg), impaired ability to walk, severe visual impairment, severe polyneuropathy, moderate to severe renal insufficiency (dialysis requirement or GFR < 10 mL/min/1.73 m^2^), and tendency to hypo- or hyperglycemia.

T2DM patients (those whose blood glucose was stable for at least 6 months via diet and/or metformin) were randomized to a walking group (n = 16) or a control group (n = 16). A total of 387 patients were screened as possible participants; 317 did not fulfil the inclusion criteria, and the remaining 70 were randomized Sixteen patients were assigned to the control group, and 18 were assigned to each of the intervention groups (walking (40 min three times a week and E-health group (pedometer-supported physical activity control (goal of 10,000 steps a day, evaluated only in our previously published study on cognitive function 2021) and 18 to the HIIT group. Only the walking group was analyzed for this publication.

The training in the HIIT group had to be discontinued because too many patients dropped out. In the other groups, twelve patients withdrew for unrelated or medical reasons after randomization and were excluded for technical reasons. Finally, the data of 16 participants from the walking group and 16 participants from the control group were evaluated. Control group patients were evaluated after 12 weeks, according to the randomization time point. The methods and results regarding cognitive function in this study population were described and published previously by our group in 2021.

The participant characteristics are presented in Table 1. Participants were excluded if they had any overt cardiac disease, participated in regular exercise (≥60 min of moderate-vigorous activity per week), or had any contraindications to exercise stress testing according to current guidelines [31] and a study by Cassidy et al. [28].

### 2.2. Ethics Statement

The institutional review board of University Witten-Herdecke (application no. 184/2015) approved the trial at 17 December 2015 and 20 July 2016. Informed consent was obtained from all participants. We recruited participants by advertising through local newspapers, social media, contacts in clinical practices, and diabetes community groups between October 2016 and January 2017.

### 2.3. Echocardiography

Echocardiographic examinations were performed by medical physicians with specific echocardiography evaluation training using a commercial ultrasound machine (Vivid E 9 Dimension^®^, General Electrics Systems (Waukesha, WI, USA)) equipped with an S3 probe (2–4 MHz). Two-dimensional assessments of the left ventricular cavity diameter, wall thickness, and mass were performed according to the criteria of the American Society of Echocardiography and the European Association of Cardiovascular Imaging [32]. Two-dimensional grayscale M-mode and B-mode images were recorded in standard parasternal short- and long-axis views as well as in apical four-, three-, and two-chamber views. Images were obtained at the level of the left ventricle and the aortic valve to assess the aortic root diameter (Ao), left atrial diameter (LA), left atrial volume (LA volume), fractional shortening (%FS), left ventricular diastolic and systolic (LVIDd and LVIDs) cavity diameters, diastolic and systolic left ventricular posterior wall (LVPWd and LVPWs) diameters, diastolic interventricular septum diameter (IVSd), systolic interventricular septum diameter (IVSs), left ventricular mass (LV mass), left ventricular end-diastolic volume (LVEDV), left ventricular end-systolic (LVESV) volume, ejection fraction (EF), and stroke volume (SV).

The left ventricular ejection fraction (LVEF) was measured via Simpson’s rule and biplane images from the apical four- and two-chamber views. The LV mass was determined via Devereux’s formula. To detect hypertrophy type, the criteria of the American Society of Echocardiography and the European Association of Cardiovascular Imaging were used [32]. We used pulsed-wave (PW) Doppler to record left ventricular velocities from the apical four-chamber view. We positioned the PW-Doppler sample volume at the tip of the mitral leaflets, with the ultrasound probe parallel to the flow stream. At this position, we measured the early (MV E-Max) and late (MV A-Max) diastolic peak velocities and their ratios (MV E/A ratio). We measured E′ as an early diastolic peak (E′ (lateral)) via Doppler tissue imaging (DTI) to record PW Doppler signals in the apical four-chamber view, with the myocardium positioned within the basal lateral wall, within 1 cm of the mitral annulus. Finally, we determined the E/E′ ratio.

Strain echocardiography is a well-established and reproducible method for assessing cardiac function [33,34]. Strain and strain rate data were recorded in apical four-, two-, and three-chamber views with a GE Vivid E9 Ultrasound Machine. Afterwards, technicians used GE Medical Systems to perform offline strain analysis via EchoPAC Dimension software (product versions 112, 1.5, 298) on a separate workstation. Apical four-, two-, and three-chamber views were recorded using a high frame rate via entry through the left ventricle in the echocardiographic plane. We intentionally chose cardiac cycles of the same length during the respiratory phase of expiration. The operator performing the analysis was not the echocardiography investigator and was blinded to the participants’ data. Semiautomatic border detection was used to identify the interface between the left ventricular walls and cavity for the analysis. When bad borders were recognized, technicians performed manual corrections to ensure correct tracing of the endocardial and epicardial border and corrected the wrong segment of the left ventricle. In this study, we recorded apical longitudinal four-, two-, and three-chamber views (to assess four-chamber strain, two-chamber strain, and three-chamber strain, respectively) via automated functional imaging (AFI). Global longitudinal strain GLS (Global strain) was calculated by averaging all regional values of peak systolic deformation determined in each segment of the three apical views (PLAX 3chamber, 4chamber view and 2chamber view) before aortic valve closure in a seventeen-segment left ventricle model. The speckle tracking method for representing the shortening of myocardial segments can describe cardiac function in its own way as a deformation parameter (strain). Furthermore, we used 2D speckle tracking to measure the longitudinal strain (LV) and peak systolic strain rate (LV longitudinal strain rate) in the apical four-chamber view. The participants’ strain data were excluded from the analysis when three or more myocardial segments could not be evaluated.

### 2.4. Spiroergometry (Cardiopulmonary Exercise Testing)

Spiroergometry is a very well-established diagnostic method for measuring cardiopulmonary fitness [35,36]. Spiroergometry was performed using a MetaLyzer^®^ metabolic cart (CORTEX Biophysik GmbH, Leipzig, Germany) with MetaSoft^®^ Studio software (version 3.9) for data acquisition and analysis.

After successful gas and volume calibration, we completed the stress test beginning at 50 watts, which was continuously increased by 25 watts every 2 min (ramp test). Abs_VO_2_max refers to absolute maximal oxygen uptake expressed in L·min^−1^ (or mL·min^−1^), whereas rel_VO_2_max refers to maximal oxygen uptake normalized to body mass, expressed in mL·kg^−1^·min^−1^. The test was completed when the participant could not maintain the predefined cadence of 80/min or if the participant was subjectively exhausted and there was no further increase in VO2max after 20 s. Spiroergometric analyses were conducted as previously described [35,37]. The ventilatory aerobic threshold (VAT) was defined as the first nonlinear increase in the ventilatory equivalent for oxygen without a simultaneous increase in the ventilatory equivalent for CO_2_. The respiratory compensation point (RCP) was defined as the simultaneous nonlinear increase in both ventilatory equivalents according to previously described recommendations. Body weight and body composition were determined via a Tanita BC-418MA segmental body composition analyser [38]. The participants were instructed to wear only comfortable shorts without any other clothing.

### 2.5. Blood Analysis, Blood Pressure, and Waist Circumference

Blood was collected via venipuncture and analysed for various parameters (MVZ Dr. Stein + Kollegen, Mönchengladbach). Serum triglycerides, total cholesterol, and high-density lipoprotein (HDL) cholesterol were measured via enzymatic methods; fasting glucose was measured via the hexokinase method. Low-density lipoprotein (LDL) cholesterol levels were determined with the Friedewald equation. Participant height (stadiometer) and weight (digital scales) were measured while he or she was barefoot to the nearest 0.5 cm and 0.1 kg, respectively.

After ≥5 min of rest, blood pressure was measured with an automated blood pressure monitor (Boso) three times while the participants were in the seated position.

Waist circumference was measured at the narrowest area, and hip circumference was measured at the widest location over light clothing using an unstretched tape metre without any pressure to the body surface and recorded to the nearest 0.1 cm. To avoid subjective error, all measurements were taken by the same investigator [39].

### 2.6. Statistical Analysis

According to G*Power (3.1.9.4), a sample size of 15 is sufficient to detect an effect size of 1.12 with a power of 0.8 and a significance level of 0.05 (two-sided *t* test for two independent groups). This sample size is also sufficient to detect an effect size of 0.82 for a within-group before-after comparison (power 0.8, significance level 0.05, two-sided *t* test for matched pairs). With the expectation of a higher dropout rate for the intervention groups, n = 18 was chosen for the intervention groups, and n = 16 was chosen for the control group.

Block randomization with a variable block length (4, 8, 12) was used. The size and order of the block lengths were random. The randomization list was generated via Stata/IC 16.1 for Unix (StataCorp, College Station, TX, USA).

Data were analyzed using random-effects linear regression (xtreg in Stata). Estimated means with 95% confidence intervals were obtained via Generalized Least Squares (GLS), which accounts for potential heteroskedasticity and autocorrelation in the errors. While these estimates are asymptotically normally distributed, the errors themselves are not assumed to be normally distributed, and no normality assumption is required for estimation or inference. The null hypothesis that a mean equals zero is tested using a Wald z-test, based on the asymptotic normality of the estimated coefficients.

Continuous data were described by mean and standard deviation.

Random-effects linear regression was used for all pre and post and between group comparisons. Post/pre ratios and Walking/Control ratios were estimated by analyzing differences in log-transformed parameters and re-transformation of these estimates. Estimated means with 95% confidence intervals were obtained via Generalized Least Squares (GLS), which accounts for potential heteroskedasticity and autocorrelation in the errors. While these estimates are asymptotically normally distributed, the errors themselves are not assumed to be normally distributed, and no normality assumption is required for estimation or inference. The null hypothesis that a mean equals zero is tested using a Wald z-test, based on the asymptotic normality of the estimated coefficients.

All statistical tests were two-sided and the significance level was set at 0.05. Stata/IC 16.1 for Unix was used for statistical analysis (StataCorp, College Station, TX, USA).

### 2.7. Experimental Protocol and Randomization

**Figure 1:**
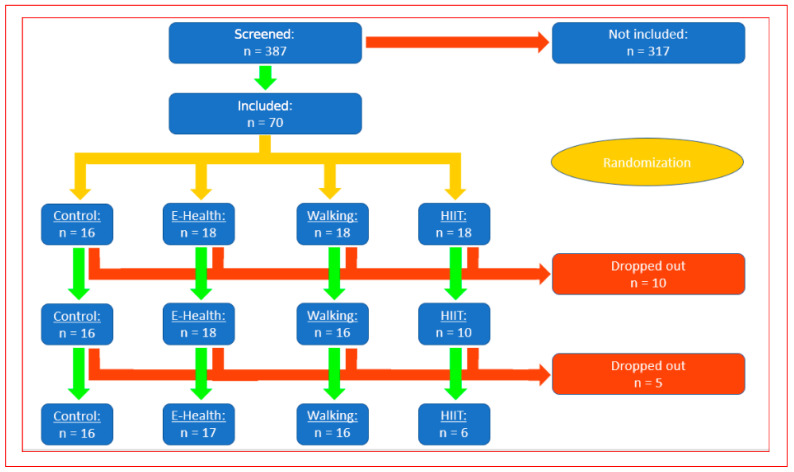
Flow diagram showing the participant numbers at each stage of the trial.

After the initial screening, spiroergometry, body composition, and blood parameters were measured at baseline and after 12 weeks of the supervised walking intervention (three times a week at 40 min) (Figure 1).

The participants in the walking group met with the instructor three times a week, and the same defined route was always completed. The goal was to increase their heart rate by at least 30 beats per minute while they were walking. The instructor set the pace so that everyone could follow without any problems and could also converse during the walk. The participants were advised that additional walking should be of a brisk nature that would leave them slightly breathless and warm but still able to talk. The walking definition we used was defined as moderate exercise (4 to 5 METS) [40,41]. Our description is comparable with that of Faggian et al. [42].

The control group was managed according to the usual standards of diabetes treatment by their general practitioner. In a 15 min conversation, the control group was advised to engage in at least 30 min of physical activity daily and to follow a healthy diet adjusted for caloric intake and blood sugar levels on the basis of their basal metabolic rate. Adherence to these recommendations was not monitored but was left to the discretion of the patients and their general practitioners.

The participants were randomized (Figure 1) into groups with a simple random list (performed by our statistical support service (P-Point)). Sealed envelopes with consecutive numbers were locked in a drawer and withdrawn in numerical order by the primary author.

## 3. Results

### 3.1. Anthropometry and Baseline Data

The baseline data of the participants in the control and walking groups are shown in Table 1, and the flow of participants throughout the study protocol is shown in Figure 1.

Ultimately, we evaluated 16 participants in the control group and 16 participants in the walking group. In the walking group, we only had 2 dropouts (18 patients were randomized, and 16 patients completed the final analysis), a married couple (one man and one woman) who could not continue with for personal reasons. After two training sessions, they stated that they ‘simply did not feel like it’ anymore. There were no dropouts in the control group. The HIIT group could not be evaluated because 10 patients were not able to complete 12 weeks of training, 3 times a week for 20 min at 80 to 90% of their maximum VO2max for personal reasons.

The analyzed groups were well matched for all baseline characteristics (Table 1). Compared with the control group, the walking group presented greater waist circumference reduction (−3 cm, CI: −4.41–1.59, *p* < 0.001) and body fat reduction (−2.74%, CI: −4.71, 0.76, *p* < 0.007). The resting heart rate changed significantly from baseline to postintervention (6.50 p/min, CI: −9.69, −3.3, *p* < 0.001) in the walking group. The results are summarized in Table 2.

### 3.2. Weight, Body Composition, and Cardiometabolic Parameters

**Figure 2:**
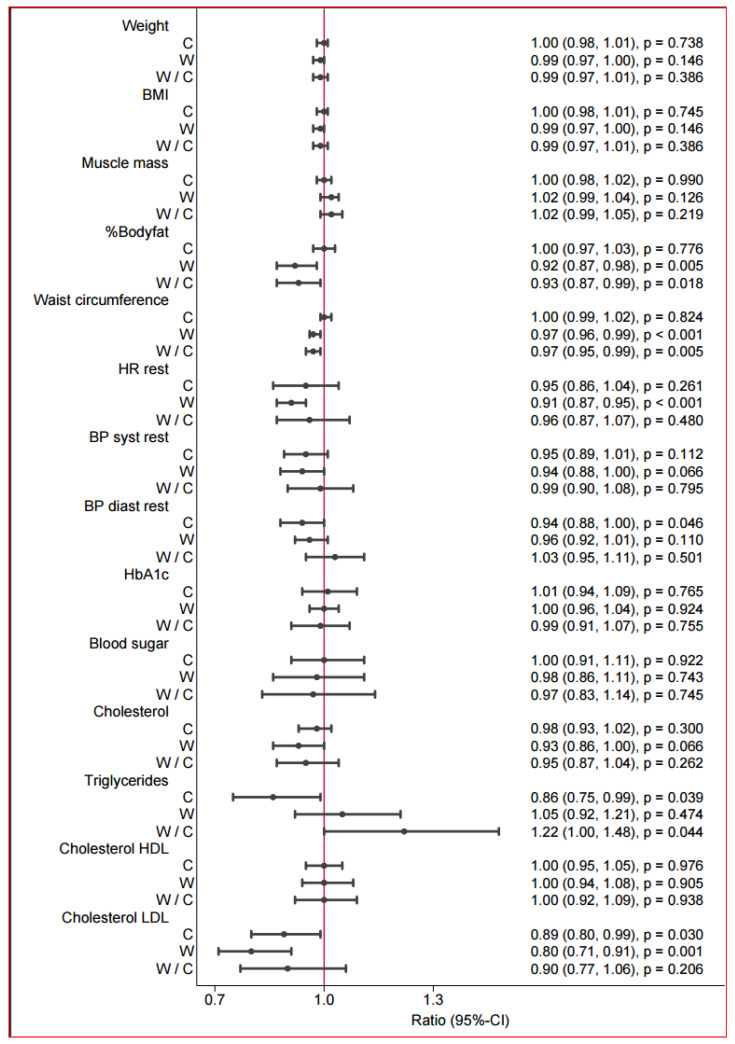
Postintervention between-group differences in anthropometric and cardiometabolic parameters. Forest plot of changes in anthropometric, hemodynamic, and metabolic variables in the control group (C) and walking group (W) and the between-group comparison (W/C). Values are shown as ratios with 95% confidence intervals (CI) and corresponding *p*-values. For C and W, the ratio represents the within-group change (follow-up relative to baseline). For W/C, the ratio represents the relative change in the walking group compared with the control group (between-group effect). Ratios < 1.0 indicate a decrease from baseline (or a lower value in W relative to C), whereas ratios > 1.0 indicate an increase. The vertical reference line at 1.0 denotes no change/no difference.

In the control group, no significant changes from baseline were observed in any of the measured parameters (Table 2 and Figure 2). In the walking group, there was no marked change in weight (according to an improvement in muscle mass). The mean difference was a nonsignificant weight loss of 1.17 kg. However, after the intervention, the walking group presented a 7.1% reduction in body weight from baseline. The waist circumference decreased significantly by 3 cm in the walking group after the intervention (3.4%). LDL levels decreased in both groups (22.7 mg/dL in the walking group and 11.8 mg/dL in the control group). Systolic blood pressure tended to decrease only in the walking group (by 8 mmHg), but the change was not significant.

### 3.3. Spiroergometry

**Figure 3:**
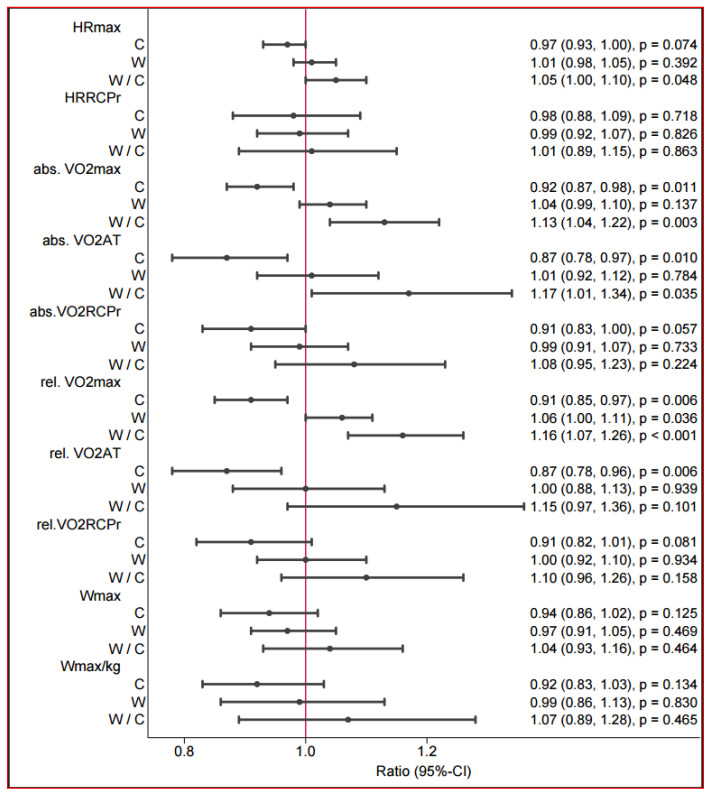
Postintervention between-group differences in spiroergometry.

The relative oxygen intake (VO2 max in mL/min/kg) changed in a positive direction only in the walking group (n = 14, 16.4 (±3.4) to 17.3 (±3.7), *p* < 0.055 (absolute estimated change in walking vs. control: 2.43 (1.03, 3.83), *p* < 0.001) (Table 3). The postintervention between-group differences are presented in Figure 3. The absolute oxygen uptake (VO2max) differed between the control and walking groups (1.13 (1.04–1.22) *p* < 0.003) (Table 3). We observed a reduction in the VO2max over 12 weeks in the control group. In the control group (control: mean difference: −4.99 bpm, 95% CI [−10.6; 0.08], *p* ≤ 0.054), the maximum heart rate tended to be lower than that in the walking group (walking: mean: 1.5 bpm, 95% CI [−2.7; 5.16], *p* ≤ 0.42). Within the groups, there was no significant difference. A significant difference was found only in the group comparison (walking vs. control: mean difference: 6.49 bpm, 95% CI [0.55; 12.42], *p* ≤ 0.032) of the maximum heart rate during exertion. At ventilatory threshold 1 (VT1), heart rate at follow-up was lower than at baseline in both groups; however, this change did not reach statistical significance.

However, the heart rate tended to decrease more strongly in the control group (control: mean difference: −2.47 bpm, 95% CI [−13.70; 6.56], *p* ≤ 0.734) than in the walking group (walking: mean difference: −1.38 bpm, 95% CI [−9.32; 6.56], *p* ≤ 0.724). In the group comparison, there was no relevant difference in heart rate at VT 1 (ventilatory threshold 1).

### 3.4. Echocardiography and Strain (Speckle Tracking)

**Figure 4:**
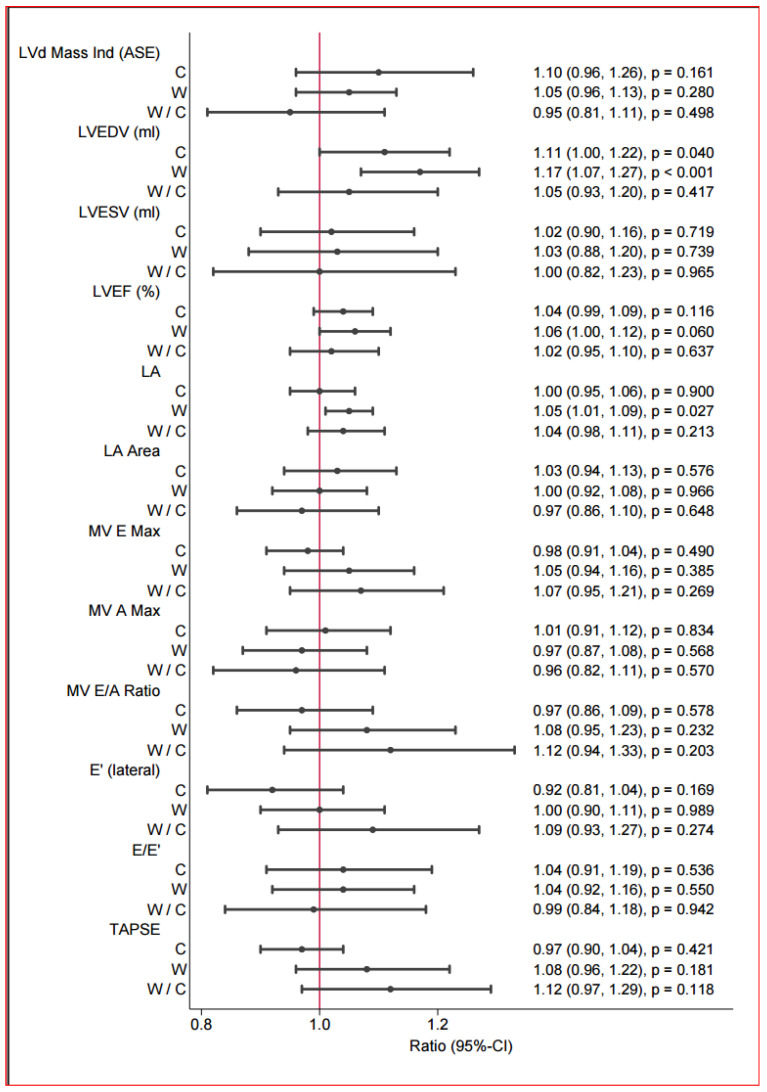
Postintervention between-group differences in echocardiography.

**Figure 5:**
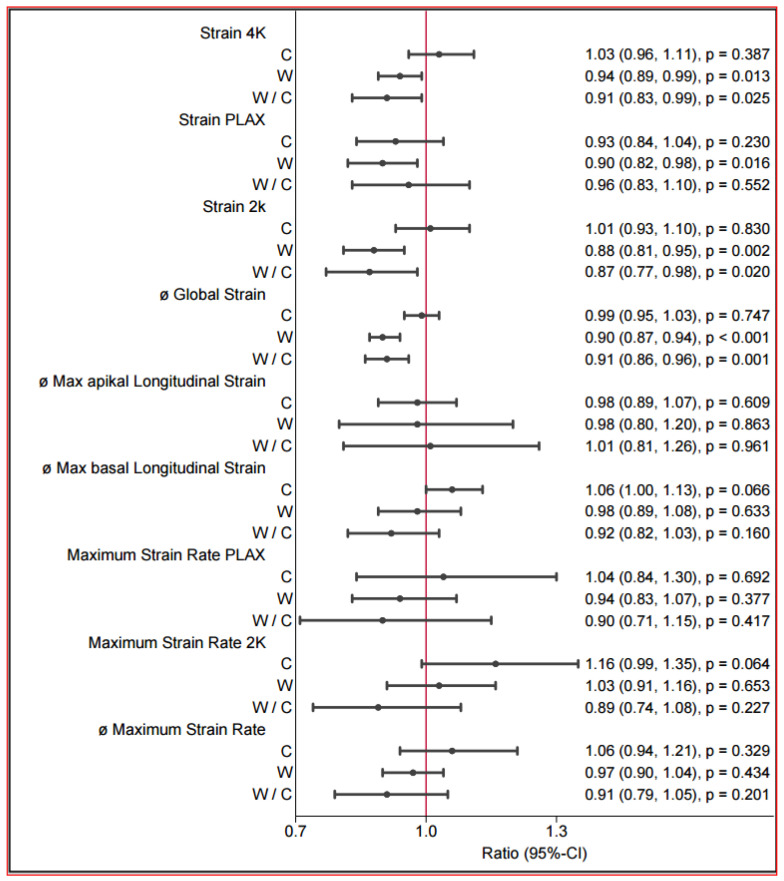
Postintervention between-group differences in strain.

The echocardiography findings revealed several changes among the groups and in the group comparisons before and after the intervention (Table 4 and Figure 4). In brief, the walking group tended towards a better ejection fraction of 3.75% (*p* < 0.068) (without significance), and there was a notable improvement in contractility, as evidenced by the speckle tracking method. Additionally, after the intervention, the size of the left atrium tended to be increased in the walking group, and the end-diastolic volume significantly improved. The increase in the LVEDV and left atrial size were additionally observed. The GLS (global strain) significantly improved myocardial contraction in the walking group (walking: mean difference: −1.92, 95% CI [−2.61; −1.24], *p* < 0.001) (Table 5 and Figure 5). In the group comparison, a significantly greater global strain was observed in the walking group than in the reference group (walking vs. reference: mean difference: 1.73, 95% CI [−2.78; −0.69], *p* ≤ 0.001). This significant improvement was supported both in the two-chamber representation (walking: mean difference: −2.25%, 95% CI [−3.60; −0.48], *p* ≤ 0.011) in the relative description and in the four-chamber representation of strain (walking: mean difference: −1.44, 95% CI [−2.50; −0.38], *p* ≤ 0.008). Improvements in diastolic function could not be evaluated.

## 4. Discussion

This study represents the first randomized trial investigating the impact of supervised walking (3 × 40 min/week; moderate intensity, ~4–5 METs) on cardiac structure and function in patients with type 2 diabetes (T2DM) using contemporary echocardiographic techniques. The primary findings indicate that a 12-week supervised walking program reduced waist circumference and percent body fat.

These anthropometric changes are clinically relevant in T2DM because central adiposity is closely linked to cardiometabolic risk and adverse cardiac loading conditions. However, improvements in myocardial function may also occur without pronounced weight loss, for example, through enhanced hemodynamic efficiency, vascular function, and cardiorespiratory fitness. Accordingly, we next focus on cardiac outcomes and report an improvement in systolic function assessed by global strain (GLS).

DM is currently among the most common health problems worldwide [43]. Whether moderate-intensity training leads to improvements in systolic function in patients with DM remains controversial [20,22,25,26,44,45]. HIIT improves systolic function in patients with DM [28,29,30]. Moderate-intensity exercise has a positive effect on cardiometabolic health [46], and walking as a moderate-intensity physical activity is among the most effective training approaches for controlling hyperglycemia [47] and reducing cardiovascular risk in DM15. Therefore, the key question of the present study was whether 12 weeks of supervised moderate exercise (three weekly walking sessions) can induce measurable improvements in anthropometric parameters and, importantly, systolic myocardial function as reflected by GLS (global longitudinal strain).

In general, there is agreement in the literature that PA, even at moderate levels, improves glycemic control and anthropometric parameters in patients with DM [48].

In addition to the evidence in diabetes cohorts, walking-based and other moderate-intensity aerobic training programs have been shown in various non-diabetic populations to improve cardiorespiratory fitness and functional capacity and, in several studies, to induce favorable changes in cardiac functional indices, particularly parameters related to left-ventricular filling and overall performance [49]. These observations support the concept that even pragmatic, low-threshold walking interventions can elicit clinically meaningful cardiovascular adaptations.

A meta-analysis by Mabire et al. [49] reviewed 22 studies analysing a walking intervention lasting a median of 12 to 16 weeks with four aerobic walking sessions per week in overweight individuals (BMI ≤ 30) aged 18 to 65 years. A reduction in BMI, an average weight loss of 2.13 kg and a decrease in waist circumference were observed. The body fat percentage decreased by an average of 1.38% after the training intervention [49]. In the publication by Fritz et al. [50], the effects of a five-time-per week Nordic walking intervention over four months were examined in participants with manifest DM, impaired glucose tolerance, and normoglycemic metabolism. Improvements in anthropometric parameters, such as reductions in BMI and body weight, were observed when participants attended at least 80% of the training sessions [50]. The results of the present study largely confirm the findings of Mabire et al. [49] and Fritz et al. [50]. Our relatively short 12-week walking intervention led to an average weight reduction of 1.2 kg, but this difference was not significant. A similar trend was observed for BMI, with an average postintervention BMI reduction of 1.2%. The present study evaluated the effects of three weekly supervised walking sessions, but weight loss could not be evaluated. However, this can be explained by the increase in muscle mass and the change in body composition. The patients in the walking group had a significant reduction in body fat percentage of 2.7%. A statistically significant reduction in body weight and BMI could be achieved through a higher training frequency and a more extended training period, as observed by Mabire et al. [49] and Fritz et al. [50]. Regular training participation seems to have substantial effects on body constitution. A publication by Di Blasio et al. [51] suggested that postmenopausal women may experience a decline in “spontaneous PA”, but participation in a supervised program can lead to successful weight reduction. An additional diet program can provide further success in achieving weight loss, and weight loss programs should incorporate both aspects: increasing PA with more walking sessions a week and adding a low-calorie diet [52]. The results of physical exertion with respect to myocardial systolic function improvement in patients with diabetes have been controversial in earlier studies [20,25,26,44,45]. This discrepancy may be related to the fact that these studies lacked direct supervision in the study design, and the populations studied in the past did not receive continuous supervision in the study [25,26]. Ofstad et al. [45] compared 44 patients receiving a multifactor intervention (advice on diet and recommended exercise, possibly with a gym membership, unsupervised over 6 months) with 45 control participants and did not detect any difference in diastolic or systolic function after 2 years. The reason for this lack of effect could be that exercise was not sufficiently monitored, exercise recommendations were not clear, and exercises were thus not correctly performed. In 2007, Loimaala et al. [44] monitored 24 T2DM patients and 24 control participants after 1 year of an exercise intervention (strength training and walking). The walking training program was not precisely described. Exercise sessions in the gym were supervised by a physiotherapist. This study did not find any changes in diastolic function (tissue Doppler measurements) or changes in peak systolic strain or the systolic strain rate. However, one can assume that the echography devices used in 2007 may have been inadequate for strain analysis. A nonrandomized study of male patients with T2DM evaluated the effects of soccer training [53] and revealed an increase in left ventricular global strain after 24 weeks of 1 h/week training (n = 12 patients/9 control participants). In randomized controlled trials that evaluated the effects of intense physical exercise [28,29,30], the authors reported a clear improvement in myocardial systolic function. Diastolic function also improves with moderate PA [27]. Cassidy et al. [28] reported an improvement in systolic function following a HIIT intervention in 14 patients in 2015. Cardiac function was assessed after 12 weeks of HIIT via magnetic resonance imaging (MRI), which revealed an increase in the stroke volume from 76 (±16) to 87 (±19) mL. Hollekim-Strand et al. [29] investigated the impact of HIIT on cardiac function in 16 individuals with T2DM and diastolic dysfunction. HIIT had greater benefits than moderate-intensity exercise did for these 16 individuals. This study highlights the potential of HIIT as a nonpharmacological strategy to improve cardiac function in individuals with T2DM and diastolic dysfunction. In the Hollekin-Strand study, HIIT improved systolic function, whereas moderate-intensity exercise training did not. A study performed by Wilson et al. [30] examined the effects of more than 3 months of HIIT on left ventricular function in 11 participants with T2DM. Compared with baseline, HIIT increased VO2peak by approximately 15% (*p* < 0.002). LVEDV and left ventricular stroke volume (LVSV) increased significantly after 3 months of HIIT. The LVSV response to exercise improved in adults with T2DM. The mitral inflow parameters and mitral annular velocities were unaffected by HIIT.

In the study presented by our working group, a 12-week supervised walking program (3 × 40 min/week, moderate-intensity exercise) led to an improvement in systolic function, and through strain technology, we developed a powerful tool for determining small systolic changes in myocardial function. The global strain (GLS) improved significantly, by approximately 11%, after 12 weeks of supervised walking training. When conventional echocardiography was considered, there was also an increase in the EF of 3.75%, although this increase was not significant (*p* = 0.061). The increase in the LVEDV and left atrial size were additionally observed could be explained by the influence of training, indicating an adaptation to regular PA.

Relative oxygen uptake (rel_VmaxO2) improved by 6%, and the absolute VO2max improved by 4.6%, even after only 12 weeks of moderate training.

If additional diet management is implemented, an improvement in stroke volume can be anticipated in patients with coronary heart disease and diabetes, even with moderate-intensity exercise. One-year lifestyle modifications, including diet and increased exercise, improve diastolic function and stroke volume in patients with coronary disease and T2DM [54]. In a relatively recent randomized study, Gulsin et al. [55] examined asymptomatic younger adults with T2DM (average age 51 years, average glycated hemoglobin [HbA1c] level of 7.3%) and matched control participants without T2DM. Unfortunately, this study revealed no improvement in diastolic function or systolic function in 22 patients in the exercise group.

The mechanisms driving this improvement are diverse [56]. Animal studies suggest that training improves myocardial function in heart failure by restoring the levels of calcium cycling proteins and myofilament sensitivity [57,58]. It is not known whether this potential discrepancy reflects a physiological difference between humans and rats [58].

Owing to patient dropout in the HIIT group (because they found it too strenuous), no comparison can be made between the walking and HIIT interventions in this study; such a discussion would be invalid and speculative. A new attempt would need to be made to find sufficient patients in a real-world, regional setting; however, these studies require substantial personal and financial effort. Comparing these results with those of an e-health group would also be complex, and the data volume and discussion of the results would exceed the scope of this publication. The E-Health group was included in a previously published study of our group on cognitive function in diabetic patients in 2021. The results of the e-health group and the discussion of the background, as well as the comparison with other e-health studies, will need to be addressed in another publication. Despite these promising findings, several limitations warrant consideration. However, no objective heart rate monitoring appears to have been implemented in the walking group. Consequently, it cannot be verified whether participants actually exercised at the targeted intensity. This represents a limitation of the study. However, we would like to note that, despite the lack of individualized heart-rate monitoring, we still observed statistically significant effects in our outcomes. This suggests that group-based walking per se—accompanied by an increase in heart rate—can produce the effects described in our study. Although the study has shown a clear effect of walking in T2DM patients, the study has a couple of limitations that should be acknowledged. The open-label design may introduce bias in participant behavior and outcome assessment. Despite careful adjustment, residual confounding cannot be excluded, including the potential influence of subclinical or undiagnosed coronary artery disease and concomitant medical therapies on myocardial function. Additionally, the study did not assess long-term prognostic outcomes, such as cardiovascular events or heart failure progression. Taken together, these factors highlight that the findings should be interpreted as hypothesis-generating and require confirmation in larger, controlled studies. In patients with type 2 diabetes, moderate-intensity walking was associated with improvements in global longitudinal strain, suggesting beneficial effects on subclinical myocardial function. However, given the study’s design limitations, small sample, and lack of prognostic endpoints, these results should be viewed as preliminary. Further research in larger, randomized, and blinded trials is needed before definitive clinical recommendations can be made.

## 5. Conclusions

In summary, on the basis of our data, even moderate-intensity PA, such as walking, can lead to improvements in systolic cardiac function.

In the present study, no significant between-group effects were observed for glycaemic or other biochemical variables (Table 2). The primary findings indicate that a 12-week supervised walking program reduced waist circumference and percent body fat and additionally an improvement of cardiac function.

Notably, the improvement in GLS (global Strain) suggests that functional cardiac adaptations can occur even in the absence of short-term changes in standard metabolic markers (in our 12 weeks study we did not observe significant biochemical changes in comparison to the control group without walking, although walking generally may improve biochemical parameters [47], this effect may become more evident over a longer observation period. In addition, our control group may also have followed, at least to some extent, the recommendations for a healthy lifestyle. Diabetic cardiomyopathy (DCM) is a severe complication of DM that causes significant symptoms and poses a global health challenge. Exercise is known to be effective at preventing and treating various chronic diseases. Recent molecular studies suggest that DCM development involves mitochondrial dysfunction, fibrosis, oxidative stress, Ca^2+^ dysregulation, and microvascular dysfunction [56]. Regular exercise can delay or even prevent complications arising from diabetes and may play a crucial role in preventing DCM and heart failure independent of its positive effects on glucose metabolism, endothelial function and inflammatory markers [59,60].

Therefore, walking is a well-recommended, affordable, simple, and universally applicable method for improving not only cardiac function but also body composition or glycemic control in patients with diabetes [61].

## Figures and Tables

**Table 1:** Group comparison of baseline characteristics.

Characteristics	Control	Walking	*p*
Age (years)	59.1 (8.5)	60.4 (5.9)	0.598
Weight (kg)	98.6 (16.5)	96.7 (15.4)	0.738
Height (cm)	170.6 (11.0)	167.7 (9.3)	0.429
BMI (kg/m^2^)	33.8 (4.4)	34.4 (4.6)	0.715
Muscle mass (kg)	60.7 (12.9)	55.4 (11.3)	0.229
Body fat (%)	35.5 (7.5)	39.5 (8.7)	0.172
Waist circumference (cm)	114.1 (11.6)	114.2 (10.4)	0.962
Heart rate (bpm)	77.6 (11.3)	76.4 (10.7)	0.774
RRsRest (mmHg)	137.9 (12.6)	140.6 (11.7)	0.544
RRdRest (mmHg)	84.4 (6.3)	83.0 (8.6)	0.608
HbA1c%	7 (1.3)	7.3 (1.3)	0.435
Blood glucose (mg/dL)	154.9 (41.9)	156.9 (39.8)	0.894
Cholesterol (mg/dL)	186.0 (29.1)	199.0 (40.0)	0.298
Triglyceride (mg/dL)	199.9 (64.9)	185.7 (114.5)	0.668
HDL (mg/dL)	50.2 (10.8)	52.4 (13.3)	0.612
LDL (mg/dL)	112.6 (33.8)	121.2 (32.7)	0.476

Abbreviations: BMI: body mass index; HDL: high-density lipoprotein; LDL: low-density lipoprotein; HbA1c%: glycosylated hemoglobin; RRs: systolic blood pressure; RRd: diastolic blood pressure.

**Table 2:** Cardiometabolic parameters: Pre–post estimated mean differences and ratios.

Parameters	Difference Pre-Post, Mean (95% CI)		Ratio Pre-Post, Mean (95% CI)					
	Control	Walking	Control	*p*	Walking	*p*	Walking/Control	*p*
Weight (kg)	−0.21 (−1.85, 1.43)	−1.17 (−2.76, 0.43)	1.00 (0.98, 1.01)	0.738	0.99 (0.97, 1.00)	0.146	0.99 (0.97, 1.01)	0.386
BMI	−0.09 (−0.64, 0.46)	−0.37 (−0.93, 0.19)	1.00 (0.98, 1.01)	0.745	0.99 (0.97, 1.00)	0.146	0.99 (0.97, 1.01)	0.386
Muscle mass (kg)	−0.12 (−1.26, 1.02)	0.82 (−0.27, 1.91)	1.00 (0.98, 1.02)	0.990	1.02 (0.99, 1.04)	0.126	1.02 (0.99, 1.05)	0.219
Body fat (%)	−0.09 (−1.04, 0.86)	−2.74 (−4.71, −0.76)	1.00 (0.97, 1.03)	0.776	0.92 (0.87, 0.98)	0.005	0.93 (0.87, 0.99)	0.018
Waist (circ./cm)	0.25 (−1.53, 2.03)	−3.00 (−4.41, −1.59)	1.00 (0.99, 1.02)	0.824	0.97 (0.96, 0.99)	<0.001	0.97 (0.95, 0.99)	0.005
HR rest (bpm)	−4.00 (−10.85, 2.85)	−6.50 (−9.69, −3.31)	0.95 (0.86, 1.04)	0.261	0.91 (0.87, 0.95)	<0.001	0.96 (0.87, 1.07)	0.480
BPs/rest	−6.75 (−15.00, 1.50)	−8.00 (−16.55, 0.55)	0.95 (0.89, 1.01)	0.112	0.94 (0.88, 1.00)	0.066	0.99 (0.90, 1.08)	0.795
BPd/rest	−4.88 (−9.78, 0.03)	−3.19 (−6.89, 0.52)	0.94 (0.88, 1.00)	0.046	0.96 (0.92, 1.01)	0.110	1.03 (0.95, 1.11)	0.501
HbA1c%	0.04 (−0.48, 0.56)	−0.03 (−0.34, 0.29)	1.01 (0.94, 1.09)	0.765	1.00 (0.96, 1.04)	0.924	0.99 (0.91, 1.07)	0.755
Blood sugar (mg/dL)	−1.50 (−16.80, 13.80)	−2.56 (−22.10, 16.97)	1.00 (0.91, 1.11)	0.922	0.98 (0.86, 1.11)	0.743	0.97 (0.83, 1.14)	0.745
Chol (mg/dL)	−4.13 (−12.48, 4.23)	−14.25 (−28.20, −0.30)	0.98 (0.93, 1.02)	0.300	0.93 (0.86, 1.00)	0.066	0.95 (0.87, 1.04)	0.262
Triglycerides (mg/dL)	−27.00 (−58.57, 4.57)	3.06 (−27.81, 33.94)	0.86 (0.75, 0.99)	0.039	1.05 (0.92, 1.21)	0.474	1.22 (1.00, 1.48)	0.044
HDL (mg/dL)	−0.06 (−2.94, 2.81)	0.63 (−3.36, 4.61)	1.00 (0.95, 1.05)	0.976	1.00 (0.94, 1.08)	0.905	1.00 (0.92, 1.09)	0.938
LDL (mg/dL)	−11.81 (−22.65, −0.98)	−22.73 (−34.95, −10.51)	0.89 (0.80, 0.99)	0.030	0.80 (0.71, 0.91)	0.001	0.90 (0.77, 1.06)	0.206

Abbreviations: Weight (kg), BMI: body mass index; HR rest: resting heart rate (unit: beats pro minute, bpm; BPs/rest: systolic blood pressure; BPd/rest: diastolic blood pressure; HbA1c%: glycosylated hemoglobin; Chol: cholesterol; HDL: high-density lipoprotein; LDL: low-density lipoprotein.

**Table 3:** Spiroergometry: Pre–post estimated mean differences and ratios (*t* test).

Characteristics	Difference Post–Pre, Mean (95% CI)		Ratio Post/Pre, Mean (95% CI)					
	Control	Walking	Control	*p*	Walking	*p*	Walking/Control	*p*
HRmax	−5.10 (−10.30, 0.10)	1.57 (−2.10, 5.24)	0.97 (0.93, 1.00)	0.074	1.01 (0.98, 1.05)	0.392	1.05 (1.00, 1.10)	0.048
HRRCPr	−3.50 (−15.64, 8.64)	−1.43 (−9.44, 6.59)	0.98 (0.88, 1.09)	0.718	0.99 (0.92, 1.07)	0.826	1.01 (0.89, 1.15)	0.863
abs_VO2max	−0.13 (−0.23, −0.02)	0.06 (−0.03, 0.16)	0.92 (0.87, 0.98)	0.011	1.04 (0.99, 1.10)	0.137	1.13 (1.04, 1.22)	0.003
abs_VO2AT	−0.14 (−0.24, −0.04)	0.01 (−0.10, 0.13)	0.87 (0.78, 0.97)	0.010	1.01 (0.92, 1.12)	0.784	1.17 (1.01, 1.34)	0.035
abs_VO2RCPr	−0.13 (−0.27, 0.01)	−0.02 (−0.15, 0.11)	0.91 (0.83, 1.00)	0.057	0.99 (0.91, 1.07)	0.733	1.08 (0.95, 1.23)	0.224
rel_VO2max	−1.50 (−2.55, −0.45)	0.93 (−0.02, 1.88)	0.91 (0.85, 0.97)	0.006	1.06 (1.00, 1.11)	0.036	1.16 (1.07, 1.26)	<0.001
rel_VO2AT	−1.60 (−2.69, −0.51)	0.00 (−1.40, 1.40)	0.87 (0.78, 0.96)	0.006	1.00 (0.88, 1.13)	0.939	1.15 (0.97, 1.36)	0.101
rel_VO2RCPr	−1.40 (−2.88, 0.08)	0.07 (−1.37, 1.51)	0.91 (0.82, 1.01)	0.081	1.00 (0.92, 1.10)	0.934	1.10 (0.96, 1.26)	0.158
Wmax	−8.90 (−22.32, 4.52)	−1.43 (−9.88, 7.02)	0.94 (0.86, 1.02)	0.125	0.97 (0.91, 1.05)	0.469	1.04 (0.93, 1.16)	0.464
Wmax/kg	−0.10 (−0.24, 0.04)	0.02 (−0.13, 0.17)	0.92 (0.83, 1.03)	0.134	0.99 (0.86, 1.13)	0.830	1.07 (0.89, 1.28)	0.465

Abbreviations: HRmax: maximal heart rate; HRRCPr: heart rate at the respiratory compensation point; abs_VO2max (mL/min): maximum rate of oxygen uptake; abs_VO2AT (mL/min): maximum rate of oxygen uptake at the aerobic threshold; abs_VO2RCPr (mL/min): maximum rate of oxygen uptake respiratory compensation; Wmax: Watt maximal during examination; Wmax/kg: maximal Watts/kg.

**Table 4:** Echocardiography: Pre–post estimated mean differences and ratios with 95% CI (*t* test).

Variables	Difference Post–Pre, Mean (95% CI)		Ratio Post/Pre, Mean (95% CI)					
	Diff. Control	Diff. Walking	Control	*p*	Walking	*p*	Walking/Control	*p*
IVSd (cm)	0.05 (−0.08, 0.18)	0.02 (−0.07, 0.11)	1.04 (0.92, 1.18)	0.531	1.03 (0.95, 1.11)	0.513	0.99 (0.86, 1.14)	0.860
LVIDd (cm)	−0.09 (−0.43, 0.26)	0.07 (−0.05, 0.20)	0.98 (0.91, 1.05)	0.553	1.02 (0.99, 1.04)	0.204	1.04 (0.96, 1.12)	0.312
LVPWd (cm)	0.13 (−0.06, 0.31)	−0.04 (−0.14, 0.06)	1.12 (0.96, 1.32)	0.160	0.97 (0.89, 1.06)	0.544	0.87 (0.72, 1.04)	0.115
RWT (cm)	0.06 (−0.02, 0.15)	−0.02 (−0.06, 0.02)	1.15 (0.94, 1.39)	0.171	0.96 (0.87, 1.05)	0.341	0.83 (0.68, 1.03)	0.088
LVd mass ind (ASE) (g/m^2^)	9.43 (−3.28, 22.14)	2.77 (−4.87, 10.41)	1.10 (0.96, 1.26)	0.161	1.05 (0.96, 1.13)	0.280	0.95 (0.81, 1.11)	0.498
LVEDV (mL)	10.07 (0.84, 19.29)	14.69 (6.35, 23.03)	1.11 (1.00, 1.22)	0.040	1.17 (1.07, 1.27)	<0.001	1.05 (0.93, 1.20)	0.417
LVESV (mL)	0.87 (−2.55, 4.29)	1.13 (−3.90, 6.15)	1.02 (0.90, 1.16)	0.719	1.03 (0.88, 1.20)	0.739	1.00 (0.82, 1.23)	0.965
LVEF (%)	2.4 (−0.61, 5.41)	3.75 (−0.17, 7.67)	1.04 (0.99, 1.09)	0.116	1.06 (1.00, 1.12)	0.060	1.02 (0.95, 1.10)	0.637
LA (cm)	−0.00 (−0.19, 0.18)	0.15 (0.02, 0.28)	1.00 (0.95, 1.06)	0.900	1.05 (1.01, 1.09)	0.027	1.04 (0.98, 1.11)	0.213
LA area (cm^2^)	0.25 (−1.01, 1.51)	−0.13 (−1.16, 0.91)	1.03 (0.94, 1.13)	0.576	1.00 (0.92, 1.08)	0.966	0.97 (0.86, 1.10)	0.648
MV E max (m/s)	−0.01 (−0.06, 0.04)	0.03 (−0.06, 0.12)	0.98 (0.91, 1.04)	0.490	1.05 (0.94, 1.16)	0.385	1.07 (0.95, 1.21)	0.269
MV A max (m/s)	−0.01 (−0.09, 0.07)	−0.03 (−0.12, 0.05)	1.01 (0.91, 1.12)	0.834	0.97 (0.87, 1.08)	0.568	0.96 (0.82, 1.11)	0.570
MV E/A ratio	−0.04 (−0.18, 0.10)	0.07 (−0.07, 0.20)	0.97 (0.86, 1.09)	0.578	1.08 (0.95, 1.23)	0.232	1.12 (0.94, 1.33)	0.203
E′ (lateral) (m/s)	−0.01 (−0.02, 0.00)	−0.00 (−0.01, 0.01)	0.92 (0.81, 1.04)	0.169	1.00 (0.90, 1.11)	0.989	1.09 (0.93, 1.27)	0.274
E/E′	0.32 (−0.84, 1.48)	0.16 (−0.85, 1.17)	1.04 (0.91, 1.19)	0.536	1.04 (0.92, 1.16)	0.550	0.99 (0.84, 1.18)	0.942
TAPSE (mm)	−0.07 (−0.25, 0.10)	0.19 (−0.08, 0.45)	0.97 (0.90, 1.04)	0.421	1.08 (0.96, 1.22)	0.181	1.12 (0.97, 1.29)	0.118

Abbreviations: IVSd: Diastolic interventricular septum diameter; LA: Left atrium diameter; LA area: Left atrial area; LVEDV: Left ventricular end-diastolic volume; LVESV: Left ventricular end-systolic volume; EF: Ejection fraction in %; MV E max: Early diastolic peak velocity; MV A max: Early diastolic peak velocity; MV E/A ratio: Diastolic peak velocity ratio; E′ (lateral): Early diastolic peak at the lateral wall; E/E′: Ratio of early diastolic mitral inflow velocity to early diastolic mitral annulus velocity; TAPSE: Tricuspid annular plane systolic excursion.

**Table 5:** Strain: Pre–post estimated mean differences and ratios with 95% CI.

Characteristics	Difference Post–Pre, Mean (95% CI)		Ratio Post/Pre, Mean (95% CI)					
	Control	Walking	Control	*p*	Walking	*p*	Walking/Control	*p*
Strain 4Ch (%)	0.51 (−0.67, 1.68)	−1.44 (−2.50, −0.38)	1.03 (0.96, 1.11)	0.387	0.94 (0.89, 0.99)	0.013	0.91 (0.83, 0.99)	0.025
Strain PLAX (%)	−0.97 (−2.60, 0.66)	−2.09 (−3.70, −0.48)	0.93 (0.84, 1.04)	0.230	0.90 (0.82, 0.98)	0.016	0.96 (0.83, 1.10)	0.552
Strain 2Ch	−0.10 (−1.42, 1.22)	−2.25 (−3.60, −0.90)	1.01 (0.93, 1.10)	0.830	0.88 (0.81, 0.95)	0.002	0.87 (0.77, 0.98)	0.020
ø Global strain	−0.19 (−1.00, 0.63)	−1.92 (−2.61, −1.24)	0.99 (0.95, 1.03)	0.747	0.90 (0.87, 0.94)	<0.001	0.91 (0.86, 0.96)	0.001
ø Max. apical longitudinal strain	−0.51 (−2.90, 1.88)	−0.77 (−4.95, 3.41)	0.98 (0.89, 1.07)	0.609	0.98 (0.80, 1.20)	0.863	1.01 (0.81, 1.26)	0.961
ø max basal longitudinal strain	0.97 (−0.03, 1.96)	−0.54 (−2.47, 1.39)	1.06 (1.00, 1.13)	0.066	0.98 (0.89, 1.08)	0.633	0.92 (0.82, 1.03)	0.160
Maximum strain rate PLAX	0.12 (−0.28, 0.53)	−0.11 (−0.33, 0.12)	1.04 (0.84, 1.30)	0.692	0.94 (0.83, 1.07)	0.377	0.90 (0.71, 1.15)	0.417
Maximum strain rate 2K	0.34 (−0.03, 0.70)	0.07 (−0.18, 0.32)	1.16 (0.99, 1.35)	0.064	1.03 (0.91, 1.16)	0.653	0.89 (0.74, 1.08)	0.227
ø Maximum strain rate	0.15 (−0.09, 0.38)	−0.06 (−0.20, 0.07)	1.06 (0.94, 1.21)	0.329	0.97 (0.90, 1.04)	0.434	0.91 (0.79, 1.05)	0.201
Maximum average strain rate 4K in 1/s	0.07 (−0.04, 0.18)	−0.11 (−0.18, −0.03)	1.06 (0.97, 1.17)	0.210	0.91 (0.85, 0.98)	0.014	0.86 (0.76, 0.97)	0.011
Maximum average strain rate PLAX	−0.02 (−0.24, 0.19)	−0.13 (−0.23, −0.03)	0.93 (0.71, 1.21)	0.579	0.89 (0.81, 0.97)	0.007	0.96 (0.73, 1.25)	0.739
Maximum average strain rate 2K	0.09 (−0.03, 0.22)	−0.02 (−0.14, 0.09)	1.10 (0.98, 1.24)	0.105	0.97 (0.88, 1.08)	0.599	0.88 (0.76, 1.03)	0.107
ø Maximum average strain rate	0.04 (−0.06, 0.15)	−0.09 (−0.16, −0.02)	1.03 (0.93, 1.14)	0.591	0.92 (0.87, 0.99)	0.021	0.90 (0.80, 1.01)	0.078

Abbreviations: Strain 4Ch: strain value in the 4-chamber view; Strain PLAX: strain value in the apical 3-chamber view; ø Global Strain: Average longitudinal Strain (from PLAX 3chamber, 4chamber View, 2chamber view, Strain 2K: strain value in the 2-chamber view).

## Data Availability

The datasets analyzed during the current study are available from the corresponding author upon reasonable request.

## Abbreviations

Abs. VO2max	Maximum rate of oxygen uptake
Abs. VO2AT	Maximum rate of oxygen uptake aerobic threshold
Abs. VO2RCPr	Maximum rate of oxygen uptake respiratory compensation point
AFI	Automated function imaging
GLS	Average longitudianal Global strain
BMI	Body mass index
DCM	Diabetic cardiomyopathy
DM	Diabetes mellitus
DTI	Doppler tissue imaging
E′ (lateral)	Early diastolic peak at the lateral wall
E/E′	Ratio of early diastolic mitral inflow velocity to early diastolic mitral annulus velocity
EF	Ejection fraction
FS	Fractional shortening
GE	General Electric
GLS	Global Strain (longitudinal Strain, average of PLAX, 4ch-view, 2ch-view)
HbA1c%	glycosylated haemoglobin
HDL	High-density lipoprotein
HIIT	High-intensity interval training
HRmax	Heart rate maximal
HRRCPr	Heart rate respiratory compensation point
IVSd	Diastolic interventricular septum diameter
IVSs	Systolic interventricular septum diameter
LA	Left atrium diameter
LA area	Left atrial area
LDL	Low-density lipoprotein
LV longitudinal strain	2D speckle-tracking longitudinal strain
LV longitudinal strain rate	2D speckle-tracking peak systolic strain rate
LVEDV	Left ventricular end-diastolic volume
LVESV	Left ventricular end-systolic volume
LVEF	Left ventricular ejection fraction
LVIDd	Left ventricular diastolic cavity diameter
LVIDs	Left ventricular systolic cavity diameter
LV mass	Left ventricular mass
LVMI	Left ventricular mass index
LVSV	Left ventricular stroke volume
LVPWd	Left diastolic ventricular posterior wall diameter
LVPWs	Left systolic ventricular posterior wall diameter
MRI	Magnetic resonance imaging
MV A Max	Late diastolic peak velocity
MV E Max	Early diastolic peak velocity
MV E/A ratio	Diastolic peak velocity ratio
PA	Physical activity
PW	Pulsed-wave
RCP	Respiratory compensation point
rel_VmaxO2	Relative rate of oxygen uptake
rel_VO2AT	Relative rate of oxygen uptake aerobic threshold
rel_VO2RCPr	Relative rate of oxygen uptake by the respiratory comp. point
RRdRest	Resting diastolic blood pressure
RRsRest	Resting systolic blood pressure
RWT	Relative wall thickness
Strain 4Ch	Strain value in the 4-chamber view
Strain PLAX	Strain value in the apical 3-chamber view
Strain 2Ch	Strain value in the 2-chamber view
TAPSE	Tricuspid annular plane systolic excursion
T2DM	Type 2 diabetes mellitus
VAT	Ventilator aerobic threshold
VT1	Ventilatory threshold 1
Wmax	Watt maximal
